# Occupational airborne exposures and asthma mortality – examining asthma as the underlying and contributing cause of death

**DOI:** 10.1186/s12890-025-03987-1

**Published:** 2025-12-01

**Authors:** Kjell Torén, Nicola Murgia, Maria Åberg, Martin Andersson, Bengt Järvholm

**Affiliations:** 1 Occupational and Environmental Medicine, School of Public Health and Community Medicine, Institute of Medicine, Sahlgrenska Academy, University of Gothenburg, Box 414, S-405 30, Gothenburg, Sweden; 2 Occupational and Environmental Medicine, Sahlgrenska University Hospital, Gothenburg, Västra Götalandsregionen Sweden; 3 Respiratory Diseases and Toxicology, University of Ferrara, Ferrara, Italy; 4 General Practice/Family Medicine, School of Public Health and Community Medicine, Institute of Medicine, Sahlgrenska Academy, University of Gothenburg, Gothenburg, Sweden; 5 Regionhälsan, Gothenburg, Västra Götalandsregionen Sweden; 6 Department of Epidemiology and Global Health, Umeå University, Umeå, Sweden

**Keywords:** Epidemiology, Relative risk, Lung diseases, Cohort, Work

## Abstract

**Background:**

The aim was to elucidate whether occupational airborne exposures increases asthma mortality.

**Methods:**

The study comprised men in the Swedish construction industry who participated in health controls in 1971–1993. Exposure was assessed using a job-exposure matrix with focus on exposures in the mid-1970s. Mortality from asthma in 1987–2015 was compared between 147,101 workers exposed to occupational airborne exposures and 26,879 foremen, using underlying and contributory cause of death from the Swedish Cause of Death Register. Mortality was assessed as relative risk with 95% confidence intervals using Poisson regression models adjusting for age, smoking, body mass index, and calendar time.

**Results:**

Among exposed workers, there were 82 deaths with asthma as the underlying cause and 212 deaths with asthma as the contributory cause vs. ten and 21 deaths in the controls. The asthma mortality based on the underlying and contributory cause was 1.92 (1.31–2.83) in relation to inorganic dust, 2.17 (1.47–3.20) in relation to fumes, 1.60 (1.04–2.47) in relation to gases, and 1.79 (1.09–2.96) in relation to wood dust. Using only the underlying cause of death showed similar mortality estimates, but with wider confidence intervals including unity.

**Conclusions:**

Occupational airborne exposures increased the asthma mortality, underscoring the need for further reduction of the airborne occupational exposures. Workers with asthma should be given information about the effects of exposure and support to decrease exposure. The study shows the importance of using both contributing and underlying cause of death in studies assessing asthma mortality risk in relation to air pollutants.

## Introduction

Asthma in adults is a fairly common disease with a prevalence of around 7–10% in the Swedish and U.S. populations. This prevalence has been more or less constant over the last decades [1–3]. Asthma-related mortality, however, has decreased over the last decades, both in the U.S. and worldwide [3, 4]. While asthma is a fairly prevalent disease in adults, mortality from asthma as the underlying cause-of-death is rare. In Sweden in 2023, 17 men aged 50–84 died from asthma as the underlying cause of death. This represents 0.06% of all deaths in this age group [5].

Asthma can cause impairment and disability and may influence the choice of occupational career. Occupational exposure to air pollutants can be much higher than the exposure in the general environment and can vary from occupation to occupation and job to job. Construction work can cause high exposure to air contaminants in some jobs. In epidemiological studies, occupational airborne exposures have been linked to increased risk of asthma [6–8]. Some studies analyzing asthma mortality from occupational exposure report increased asthma mortality in dust and sensitizer-exposed occupations, as well as in relation to specific dust exposures [9–14]. Still, the contribution of occupational exposures as a cause of severe asthma and asthma mortality is probably underrecognized [12]. Asthma mortality reflects both the total burden of asthma and the burden of poorly controlled asthma [15]. Hence, an asthma death could be regarded as a very severe exacerbation of asthma, which may have been triggered by airborne occupational exposures [16]. Furthermore, persons with severe asthma can have chronic airflow limitation which may increase the probability of developing chronic obstructive pulmonary disease (COPD).

The first aim of the present study was to elucidate whether occupational exposures to a broad group of air contaminants such as vapors, gases and irritants, dusts and fumes in the Swedish construction industry increase mortality from asthma by analyzing both underlying and contributory causes of death. The second aim was to analyze the additional value of using contributing causes of death for risk in the asthma risk analyses.

## Methods

From 1968 to 1993, Swedish construction workers were invited to medical examinations at the occupational health service. Although this program was voluntary, around 80% of eligible workers participated at least once [17]. Data from the health examinations, including occupational title, smoking habit, blood pressure, height and weight were recorded in a central database established in the early 1970 s, as previously described [18, 19]. The database includes records of medical examinations performed during the period 1971–1993.

The original cohort consisted of 354,718 men and 34,414 women; the present analysis was restricted to men. Men who were examined for the first time below the age of 15 or above age 65 (the previous age of retirement) were excluded. Furthermore, men for whom data on calculation of body mass index (BMI) were missing, or whose BMI was < 18.5 kg/m^2^ or > 35.0 kg/m^2^ were excluded. Because of changes in the Swedish system of classification of deaths we limited the analysis to deaths from 1987. We restricted the analysis to ages between 50–84-year-olds as death from asthma before the age of 50 is rare and identifying the cause of death may be less precise at high ages. Men who had died or emigrated before 1987 were excluded.

Through the men’s personal identity number and a linkage with the Swedish Cause of Death Register it was possible to identify subjects who had died between 1987 and 2015. Both underlying causes and contributory causes of death were registered. We used the three-digit codes of the International Classification of Diseases (ICD), 10th revision (ICD-10) and 9th revision (ICD-9) for the diagnoses. The Swedish Cause of Death Register used ICD-9 during 1987 to 1996 and ICD-10 from 1997 [20, 21]. The codes we used are summarized in Table 1.

**Table 1:** Used codes from the International Classification of Diseases (ICD), 10th revision (ICD-10) and 9th revision (ICD-9)

Diseases	ICD codes	
	ICD-9	ICD-10
Asthma	493	J45 - J46
Chronic obstructive pulmonary disease (COPD)	492, 496	J43 - J44
Chronic bronchitis	491	J41 - J42
Ischemic heart disease (IHD)	410–414	I20 – I25
Pneumonia	481–483, 485–486	J13 – J16, J18
Lung cancer	162	C34

Construction workers have a varying exposure to air contaminants. We used the occupational title given at their first health examination to estimate their exposures. A job exposure matrix (JEM) was developed for selected exposures, as previously described [18]. The JEM was based on exposure estimations by industrial hygienists from the 1970’s. For this study, the exposures were merged into four broad categories, namely exposure to inorganic dust (asbestos, cement dust, concrete dust, man-made mineral fibres, or quartz); gases and irritants (organic solvents, epoxy resins, or diisocyanates); fumes (metal fumes, asphalt fumes or diesel exhaust); and wood dust. About two-thirds of the jobs were classified as having exposure to these exposures [18]. In the analysis we merged the exposures inorganic dust, gases and irritants, fumes and wood dust into a category comprising of “any” exposure.

Foremen were selected as controls. Foremen often begin working as blue-collar workers in the construction industry but their life-time exposure to air pollutants is lower than for blue-collar workers. Hence, the study population included men who according to their first job title were exposed to inorganic dust, fumes, gases or irritants, or wood dust (*n* = 147, 101) along with foremen (*n* = 26,879) with some exposure (Table 2).

**Table 2:** Basic data on the cohort of Swedish male construction workers followed between 1987 and 2015

Occupational exposures^*^	Subjects (*N*)	Body mass index (%)			Year of birth, mean	Smoking habits (%)				
		18.5–24.9 kg/m^2^	25.0–29.9.0.9 kg/m^2^	30.0–35.0 kg/m^2^		Never	Former	Light	Heavy	Un-known
Controls	26,879	61.0	34.6	4.5	1939.9	39.7	18.0	20.7	13.5	8.2
Inorganic dust	77,678	61.4	34.4	5.2	1941.3	31.8	17.2	28.8	14.7	7.6
Gases and irritants	41,248	67.5	29.0	3.6	1944.0	37.7	15.7	26.0	14.5	6.1
Fumes	64,072	58.0	36.1	5.9	1941.5	30.3	17.3	28.6	15.4	8.4
Wood dust	16,507	66.4	29.9	3.2	1942.6	39.8	16.1	25.0	12.3	6.7
Any exposure	147,101	62.3	33.0	4.7	1942.1	34.0	16.6	27.6	14.5	7.3

*Note that an individual can appear in more than one exposure category

Smoking habits were categorized based on information from the first health examination. If information on smoking was lacking in the records of the first examination, we used information from the second or third visit. For some subjects, smoking habits were unknown. The current smokers were stratified according to smoking habits at the time of examination into light (< 15 cigarettes per day) or heavy smokers (≥ 15 cigarettes per day). Based on smoking habits at baseline, subjects were consequently classified as “never-smokers”, “ex-smokers”, “light smokers”, “heavy smokers” or “persons with unknown smoking habits”.

### Statistics

Relative risks (RRs) were estimated using Poisson regression models, with mortality from asthma as the dependent variable. The analyses were restricted to subjects 50 to 84 years of age, and the subjects were followed from 1987 or from the year after the first examination until death or until 2015, whichever came first. The models were adjusted for smoking (five categories, non-smoker, ex-smoker, moderate smoker < 15 cig/day, heavy smoker 15 + cig/day, and unknown smoking habits), age (four categories: 50–59, 60–69, 70–79, 80–84 years), BMI (three categories: 18.5–24.9, 25–29.9.9 and 30–34.9 kg/m^2^), and period of observation (three categories: 1987–1996, 1997–2006, 2007–2015), all as categorical variables. The 95% confidence intervals (CIs) of the RRs were calculated using Wald estimates. The SAS^®^ program PROC GENMOD was used for the calculations (SAS Institute, Cary, NC, USA).

## Results

Data on the studied men are presented in Table 2. Among the 147,101 exposed men, 52.8% were exposed to inorganic dust, 28.0% to gases and irritants, 43.6% to fumes, and 11.2% to wood dust. There was an overlap between the exposures, especially between inorganic dust and gases and irritants. Table 2 shows smoking habits, BMI-groups and year of birth.

The cohort was followed from 1987 to 2015, and during the follow-up time there were 82 deaths with asthma as the underlying cause-of-death among the exposed workers, and ten among the controls (Table 3). For the same period, there were 212 deaths with asthma as the contributing cause of death among the exposed workers, and 21 deaths with asthma as the contributing cause of death among the controls (Table 3).

**Table 3:** Mortality, assessed as relative risks, from asthma in 50–84-year-old Swedish construction workers, 1987–2015. Poisson regression analysis, adjusted for smoking, age, Body Mass Index (BMI), and calendar period^*^

Occupational exposure	Asthma as underlying cause of death		Asthma as contributing cause of death		Asthma as either underlying or contributory cause of death	
	*N*	RR (95% CI)	*N*	RR (95% CI)	*N*	RR (95% CI)
Controls	10	1	21	1	31	1
Inorganic dust	45	1.61 (0.81–3.20)	120	2.08 (1.30–3.31)	165	1.92 (1.31–2.83)
Gases and irritants	20	1.53 (0.71–3.29)	43	1.64 (0.97–2.79)	63	1.60 (1.04–2.47)
Fumes	39	1.80 (0.89–3.61)	107	2.34 (1.46–3.75)	146	2.17 (1.47–3.20)
Wood dust	8	1.22 (0.47–3.15)	25	2.09 (1.16–3.77)	33	1.79 (1.09–2.96)
Any exposure	82	1.62 (0.84–3.13)	212	2.05 (1.31–3.21)	294	1.91 (1.32–2.76)

*Three periods; 1987–1996, 1997–2006, and 2007–2015

*N* number of deaths, *RR * relative risk, *CI* confidence interval

Analyzing mortality using asthma as the underlying or contributing cause of death showed increased mortality for any occupational exposure (RR = 1.91, 95% CI 1.32–2.76); inorganic dust (RR = 1.92, 95% CI 1.31–2.83); gases and irritants (RR = 1.60, 95% CI 1.04–2.47); fumes (RR = 2.17, 95% CI 1.47–3.20), and wood dust (RR = 1.79, 95% CI 1.09–2.96) (Table 3). Analyzing the mortality risk for asthma based only on the underlying cause of death showed similar mortality estimates but wider confidence intervals including unity (Table 3). The highest mortality estimate was for fumes 1.80 (95% CI 0.89–3.61) (Table 3). Analysis of only the contributory cause of death likewise showed similar mortality estimates with the highest estimate being for fumes 2.34 (95% CI 1.46–3.75) (Table 3). Table 4 presents the crude models adjusting only for age, with similar results to the more adjusted models.

**Table 4:** Mortality, assessed as relative risks, from asthma in 50–84-year-old Swedish construction workers, 1987–2015, 50–84 years of age. Poisson regression analysis, adjusted for only age

Occupational exposure	Asthma as underlying cause of death		Asthma as contributing cause of death		Asthma as either underlying or contributory cause of death	
	*N*	RR (95% CI)	*N*	RR (95% CI)	*N*	RR (95% CI)
Controls	10	1	21	1	31	1
Inorganic dust	45	1.79 (0.90–3.56)	120	2.29 (1.44–3.64)	165	2.13 (1.45–3.23)
Gases and irritants	20	1.76 (0.83–3.77)	43	1.79 (1.06–3.02)	63	1.78 (1.16–2.74)
Fumes	39	1.89 (0.95–3.80)	107	2.50 (1.56–3.98)	146	2.30 (1.56–3.39)
Wood dust	8	1.61 (0.64–4.09)	25	2.37 (1.33–4.24)	33	2.13 (1.30–3.48)
Any exposure	82	1.81 (0.94–3.48)	212	2.22 (1.42–3.48)	294	2.09 (1.44–3.02)

*N* number of deaths, *RR* relative risk, *CI* confidence interval

There were fewer cases among the never-smokers, and we only present the analysis for mortality risk using the combination of either the underlying or the contributory cause of death. The mortality estimates for all exposures were similar, but slightly lower than for the whole cohort, and all confidence intervals included unity. The highest mortality in never-smokers was observed in relation to exposure to fumes (2.04, 95% CI 0.97–4.28), followed by wood dust (1.76, 95% CI 0.68–4.60), inorganic dust (1.74, 95% CI 0.83–3.63), gases and irritants (1.39 (95% CI 0.60–3.23), and any exposure (1.77, 95% CI 0.88–3.56).

Among the deceased persons with asthma as the contributing cause of death, the most common underlying cause of death was ischemic heart disease (IHD) (22.4%), followed by COPD (5.2%) (Table 5). Of note, there was no cases where COPD was the underlying cause-of death among the controls with asthma as the contributing cause of death (Table 5).

**Table 5:** Underlying cause of death in men with asthma as contributing cause of death

Occupational exposures	*N*	Underlying cause of death				
		COPD	Chronic bronchitis	IHD	Lung cancer	Pneumonia
Controls	21	*N* = 0	*N* = 0	14.3% (*N* = 6)	2.4% (*N* = 1)	0
Inorganic dust	120	4.2% (*N* = 5)	< 1% (*N* = 1)	21.7% (*N* = 52)	1.7% (*N* = 4)	0
Gases and irritants	43	9.4% (*N* = 4)	*N* = 0	18.6% (*N* = 16)	1.2% (*N* = 1)	1.2% (*N* = 1)
Fumes	107	2.8% (*N* = 3)	< 1% (*N* = 2)	22.4% (*N* = 48)	1.4% (*N* = 3)	0
Wood dust	25	4.0% (*N* = 1)	0	22.0% (*N* = 11)	0	0
Any exposure	212	5.2% (*N* = 11)	1.0% (*N* = 4)	22.4% (*N* = 90)	1.2% (*N* = 5)	< 1% (*N* = 1)

*N* Number of deaths, *COPD* chronic obstructive pulmonary disease, *IHD* ischemic heart disease

## Discussion

The first conclusion from the present study is that airborne occupational exposure increases the mortality from asthma. These results give additional support to the existing evidence that occupational airborne exposures are important risk factors for severe asthma and adult asthma exacerbations.

The second conclusion is that the combined use of the contributory and underlying causes of death increases the power of the study because this way more cases are included, which increases the precision of the risk estimates. This will give us a better understanding of the impact of occupational air contaminants on mortality risk. Hence, we recommend combining the contributory and the underlying cause of death in epidemiological risk studies of on asthma.

We consider that these results to have high and external validity, although the risks may be underestimated. Most of the included controls had previously worked as construction workers and even as foremen were to some extent exposed to occupational air pollutants. Furthermore, construction workers must be physically fit to be able to work at a construction site. Therefore, persons with asthma in adolescence may choose other jobs. The relative risks were similar between the different exposures, which probably reflect that there is a mixture of exposures at a construction site during the study period. Overall, the findings indicate almost a doubled risk of asthma deaths due to air pollutants among workers in the Swedish construction industry.

We observed in a previous study that occupational air contaminants, especially organic dust, is associated with severe exacerbations of asthma [22]. The present results underscore that workers with asthma are susceptible to airborne exposure to air contaminants at the workplace with increased risk for severe exacerbations, even death. This means that workers with asthma should strongly be recommended to decrease their exposure to air contaminants. Hence, clinicians treating patients with asthma, especially severe and difficult-to-treat asthma, must take an occupational history to identify possible exposures to irritants and sensitizers [3, 23]. The current results are based on exposure assessments in the Swedish construction industry during the 1970 s, but we consider these results applicable also to other workplaces (external validity) with exposure to dust and similar air pollutants.

Previous studies have shown that considering only the underlying cause of death will underestimate the true prevalence of asthma and the actual asthma-related mortality [24, 25]. When asthma mortality was analyzed only using underlying cause of death, about half of all asthma deaths were missed in a study from the UK [26]. For the broader disease entity, obstructive lung disease, it was noted in the Tucson study that even with antemortem evidence of severe obstructive lung disease, obstructive lung disease was only reported on the death certificates as the underlying cause of death in 6.6% and as contributory cause of death in 26.0%, indicating that obstructive lung disease is underreported on death certificates [27, 28].

Studies of other diseases have also indicated the benefits of using both underlying and contributing causes of death in mortality studies [29, 30]. It seems from the literature that we should increase the use of contributing cause of death in epidemiological mortality studies of risk factors. For asthma, there may be different mechanisms. One mechanism is that asthma mortality, considering asthma as either the underlying or the contributory cause of death is a proxy for the prevalence of asthma. Another mechanism is, as previously discussed, that asthma death may be the effect of recent events, for instance a period with high exposure to occupational air pollutants reflecting a very severe exacerbation of asthma regardless of the underlying etiology of the asthma disease. Hence, asthma presented as the underlying cause of death may more reflect a death due to an exacerbation of the asthma, while asthma as the contributing cause may more reflects the occurrence of asthma.

In our study, ischemic heart disease was the most common underlying cause (22.4%) among the exposed workers when asthma was the contributing cause of death, followed by COPD (5.2%) and lung cancer (1.2%). In a British study where obstructive lung disease was mentioned as a contributory cause of death, the most common underlying causes of death among men were ischemic heart disease (18.0%), respiratory cancer (3.7%) and bronchopneumonia (1.9%) [31]. In another British study comprising both men and women, where asthma was the contributing cause of death, the underlying cause was ischemic heart disease in 27%, COPD in 7.8% and lung cancer in 4.7% [26]. Of note, in our cohort of men, among those with asthma as contributing cause of death, there were no cases with COPD as underlying cause of death among the controls compared with 5.2% among the exposed men. This may reflect that exposure to dust and fumes is also a risk factor for COPD [19]. There was also less IHD (as underlying cause of death) among the controls compared with the exposed workers.

The present study has several limitations. An important limitation is the low power due to few cases with asthma as the underlying cause of death, which results in estimates with wide confidence intervals often including unity. Hence, the additional use of contributory cause of death, considerably increased the number of exposed asthma cases. The exposure assessments for the JEM were based on exposure estimations from the 1970 s, while different exposure levels may have occurred earlier or later. It originated from an extensive exposure assessment project engaging several industrial hygienists with experience from the construction industry, and the assessors were not informed about the occurrence of asthma which otherwise may have biassed the assessments. As mentioned, the controls in our study, the foremen, had some exposures to air pollutants, albeit lower than in the exposed group, as there is some dust exposure at most construction sites. Furthermore, our exposure assessment is based on the job title at the first health control and previous jobs among foremen may have included higher exposures. This may have caused an underestimation of the risks. Comparisons between exposed workers may include differences both in the type of dust and in levels of dust and between pre- and post-examination exposure. Due to low power, we restricted the analyses to the broader groups of exposure, inorganic dust, gases and irritants, fumes and wood dust, and abstained from using the more specific exposures. Another limitation is that we have only information about the exposure from the year of the first examination, and thereafter we have assumed the same occupational exposure. Hence, we have not performed time-dependent analyses. An important limitation is that the study only comprises men and, hence, lacks external validity for women.

The classification of smoking habits in the study was not time-dependent but we did differentiate at baseline between smokers with high and low daily tobacco consumption. Still, the results for the whole group may be criticized, as the exposed group may have had different cumulative exposure to tobacco smoke, i.e. different pack-years.

The strengths of our study are that the exposure is based on expert assessment before analysis of the outcomes, the outcomes are underlying and contributory causes of death, and the design is prospective. We have also presented crude models with only adjustments for age showing similar results as the more adjusted models, and we do not present risk estimates for the potential confounding variables used for adjustments. This is in accordance with recent guidelines and supports the validity of the results [32, 33]. The study is large, covering almost all Swedish construction workers during the period of the health examinations. Having an internal control group, foremen, limits the influence of selection bias, as non-exposed construction workers have a similar socioeconomic background. Furthermore, work in the construction industry usually requires some physical fitness and persons with severe asthma in adolescence or other weaknesses may not be fit enough for employment in the construction industry.

In conclusion, occupational exposure to airborne contaminants in the Swedish construction industry increased the asthma mortality, underscoring the need for further reduction of the airborne occupational exposures to decrease the risk of adult asthma and asthma exacerbations. Exposed workers with asthma should be provided with information about the effects of exposure and be given support to reduce exposure. The study shows the importance of using both contributing and underlying cause of death in analysis of the relation between occupational air contaminants and asthma mortality.

## Data Availability

The dataset used during the current study is available from the corresponding author on reasonable request. As the study outcomes are based on matching with national registers, the availability requires ethical approval from a Swedish ethics committee.

## Abbreviations

COPD: Chronic obstructive pulmonary disease
IHD: Ischemic heart disease
BMI: Body mass index
ICD: International Classification of Diseases
JEM: Job exposure matrix
RR: Relative risk
CI: Confidence interval
